# Adverse Reactions after Booster SARS-CoV-2 Vaccination Have Less Impact on Antibody Response than after Basic Vaccination Scheme

**DOI:** 10.3390/vaccines11010182

**Published:** 2023-01-15

**Authors:** Andrea Kanizsai, Laszlo Zavori, Tihamer Molnar, Margit Tőkés-Füzesi, Zoltan Szalai, Janos Berecz, Reka Varnai, Zoltan Peterfi, Attila Schwarcz, Peter Csecsei

**Affiliations:** 1 Department of Dentistry, Medical School, University of Pecs, 7624 Pecs, Hungary; 2 Salisbury NHS Foundation Trust, Salisbury SP2 8BJ, UK; 3 Department of Anaesthesiology and Intensive Care, Medical School, University of Pecs, 7624 Pecs, Hungary; 4 Department of Laboratory Medicine, Szigetvár Hospital, 7900 Szigetvár, Hungary; 5 Department of Internal Medicine, Szigetvár Hospital, 7900 Szigetvár, Hungary; 6 Szigetvár Hospital, 7900 Szigetvár, Hungary; 7 Department of Primary Health Care, Medical School, University of Pecs, 7624 Pecs, Hungary; 8 Department of Infectology, 1st Depertment of Internal Medicine, Medical School, University of Pecs, 7624 Pecs, Hungary; 9 Department of Neurosurgery, Medical School, University of Pecs, 7624 Pecs, Hungary

**Keywords:** SARS-CoV-2, booster vaccination, adverse reactions

## Abstract

Background: It is known that adverse reactions following SARS-CoV-2 vaccinations show a positive correlation with the subsequent antibody titer. However, it is not clear how the adverse reactions following the booster vaccination are related to the antibody levels that can be measured after a 3rd dose. The primary goal of this study was to investigate whether the adverse reactions following the booster vaccination show a correlation with subsequent antibody levels. Methods: Adverse reactions occurring within 7 days after the 3rd vaccination were recorded and the anti-SARS-CoV-2 spike protein immunoglobulin (Ig) level in the venous blood was measured on post-vaccination 14th, 60th and 120th days. Results: A total of 218 volunteers were included in the study. Main findings: (i) The adverse reactions that appeared after the booster dose did not show a positive correlation with the subsequent antibody level, except a correlation in the case of fever; (ii) there were more symptomatic patients in the group receiving heterologous booster vaccine, (iii) fever after the 2nd dose was independently associated with a reduction in the likelihood of COVID-19 positivity after the booster dose. Conclusion: No adverse reactions, but fever showed a correlation with the antibody level after the booster SARS-CoV-2 vaccine.

## 1. Introduction

Severe acute respiratory syndrome coronavirus 2 (SARS-CoV-2) infection has affected hundreds of millions of people in a worldwide epidemic. In response to the epidemic, mRNA-based vaccines were the first to appear, which have a very high efficiency of over 90% [1,2]. The mRNA-based vaccines are well tolerated, adverse reactions after vaccination are common, but usually mild and self-limiting [3]. The effect of the vaccines decreases over time, so in order to restore effectiveness, a booster dose was considered necessary. The adverse reactions experienced after the booster dose are similar to those observed after the 2nd vaccination [4]; however, systemic adverse reactions were significantly higher after heterologous booster doses (17%) than after homologous booster schedules, where less than 12% of participants were affected [4]. Several other papers—including one of our previous studies—also underline that adverse reactions following vaccination correlate with post-vaccination antibody levels [5,6,7]. Individuals who have recovered from COVID-19 showed increased reactogenicity after vaccination and had higher RBD-IgG titers compared to those who were vaccinated but did not undergo a COVID infection [6]. The COVID infection represents a more complex antigenic exposure to the body than a vaccine developed only against the spike protein. Patients who have already experienced COVID infection have been shown to have increased immunogenicity after vaccination and produce higher antibody titers than vaccinated but previously uninfected individuals [8,9]. A previous infection can be responsible for the immune boost and can have similar effects to a vaccine dose [10]. When supplemented with a different type of (mRNA) vaccine, it can mimic the effects of a heterologous vaccination regimen. Based on this, we planned to investigate whether the correlation between the adverse reactions experienced after the booster vaccination and the antibody levels measured 14, 60 and 120 days later would change compared to the clear positive correlation observed after the second vaccination in our previous study [5]. The primary goal of our present study was to investigate whether the adverse reactions following the booster dose show a correlation with the subsequent antibody levels. Our secondary goal was to examine which factors influence COVID-19 positivity after the 3rd vaccination.

## 2. Methods

### 2.1. Study Design

The study population is identical to the volunteer group of our previous study, who were health care workers of Szigetvár Hospital and received 2 doses of BNT162b2 mRNA (Pfizer/BioNTech, Comirnaty) vaccination between 27 January and 9 May 2021. The adverse reactions following the vaccinations were registered and the anti-SARS-CoV-2 spike Ig level was measured at 14, 30, 60, 90, 120, 150 and 180 days after the 2nd vaccination. Clinical data and details about adverse reactions were collected from medical records and dedicated questionnaire [5]. After the booster vaccination schedule became available, we contacted the volunteers again to participate in our follow-up study. Local and systemic solicited adverse reactions after the booster dose were recorded. This corresponds to the methodology used in our previous study. Based on the adverse reactions occurring within 7 days after vaccination, two groups were created: (i) symptomatic (side effects within 7 days after the 3rd dose) vs. asymptomatic (no reported side effects 7 days after the 3rd dose). We recorded the type of booster vaccine (heterologous vs. homologous schedules) and any COVID-19 positivity within 3 months following the booster dose.

### 2.2. SARS-CoV-2 Antibody Measurements

SARS-CoV-2 Ig titers were measured in all volunteers on days 14, 60 and 120 after the 3rd vaccination. Blood samples for antibody measurements were drawn from volunteer health care workers via venepuncture with a 21-gauge needle into a closed system blood sampling serum separator tube without anticoagulant (Vacuette ^®^, Greiner Hungary LTD, Mosonmagyarovar, Hungary). Samples were tested for quantitative IgG antibodies against SARS-Cov-2 spike protein in peripheral blood on fully automated benchtop Access2 analyzer (Beckman Coulter Hungary LTD) according to the manufacturer instructions. Antibodies to the SARS-CoV-2 spike protein were determined with Beckman-Coulter Access SARS-CoV-2 IgG (1st IS) assay (Beckman Coulter Hungary LTD). The test measures IgG antibodies recognizing the receptor-binding domain (RBD) of the spike protein of SARS-CoV-2 virus. The Access SARS-CoV-2 IgG (1st IS) assay is a two-step paramagnetic particle, chemiluminescent immunoassay. Briefly, the patient sample is added to a reaction vessel with a buffer, and paramagnetic particles coated with recombinant SARS-CoV-2 protein specific for the receptor binding domain (RBD) of the S1 protein. After incubation in a reaction vessel, materials bound to the solid phase are kept in a magnetic field while unbound materials are washed away. A monoclonal anti-human IgG alkaline phosphatase conjugate is added and the conjugate binds to the IgG antibodies captured on the particles. The unbound conjugate is removed in a second separation and wash step. A chemiluminescent substrate is added to the vessel and light generated is measured with a luminometer. The light production is directly proportional to the concentration of SARS-CoV-2 IgG antibody against RBD domain in the sample. The amount of analyte in the sample is determined from a stored, multi-point calibration curve. The results are given in IU/mL, that are correlated with the First WHO International Standard Anti-SARS-CoV-2 Immunoglobulin (Human), NIBSC code, 20/136, in BAU/mL (BAU: Binding Antibody Units). The conversion of IU/mL concentrations to BAU/mL, can be done by multiplying IU/mL by multiplication factor 1. The results were interpreted as follows: cut-off index <30 IU/mL as non-reactive and reactive >/=30 IU/mL.

### 2.3. Ethics Statement

This study was conducted in conformity with the principles of the Declaration of Helsinki and approved by the Hungarian National Public Health Centre (40576-8/2021/EÜIG). Written informed consent was provided by all participants before enrollment in the present study.

### 2.4. Statistical Analysis

Data analysis was performed using SPSS (version 26; IBM, Armonk, NY, USA). Descriptive statistics were expressed as percentages for categorical data or mean ± standard deviation (SD) and median (interquartile range) for normally or non-normally distributed continuous data, respectively. The between-group difference was calculated with χ^2^, Fisher’s exact, Mann–Whitney U, and Kruskal–Wallis tests in line with suitability. To explore the independent predictors of S-Ig level and SARS-CoV-2 positivity after booster dose, a binary logistic regression was used. The odds ratio (OR) and 95% confidence interval 112 (95% CI) were calculated. The significance level was considered as *p* < 0.05.

## 3. Results

### 3.1. Participants Characteristics

**Figure 1:**
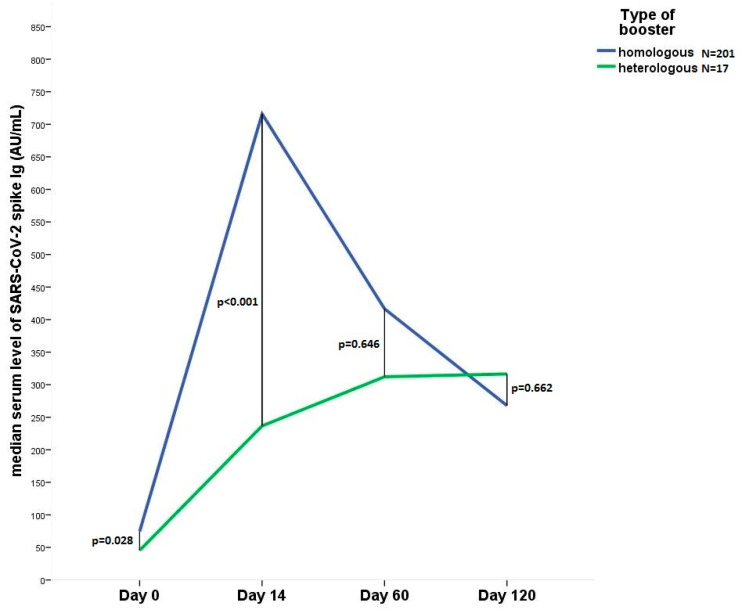
Line diagram shows the change in the median anti SARS-CoV-2 IgG level 14, 60 and 120 days after heterologous (N = 17) or homologous (N = 201) booster vaccination. Day 0, immediately before 3rd dose.

Collectively, 218 patients were enrolled and underwent blood sampling before and after (Day 14, 60, 120) the 3rd dose of SARS-CoV-2 vaccination. The initial cohort consisted of 383 volunteers [5], 218 volunteers from the previous cohort were included in the present study. Reasons for dropout: discontinued study (n = 101), loss to follow-up (n = 25), withdrawal of consent (n = 14), physician decision (n = 3), dead (n = 2), other (n = 20). The mean age was 47.6 years, with a prevalence of females (79%). The time difference between the 2nd and 3rd vaccine doses was 249 ± 44 days. A total of 35% (N = 77) of participants experienced adverse reactions following the 3rd vaccination. A total of 28% (N = 62) of the participants in the study experienced SARS-CoV-2 infection before the 3rd dose, 25% (N = 54) of them became positive after the 3rd dose. The median serum SARS-CoV-2 spike Ig level was 72 AU/mL (IQR: 36–135) before the 3rd dose, 639 (424–1100) at 14 days, 413 (215–742) at 60 days, and 268 (128–594) at 120 days after the third dose. In the symptomatic group, the proportion of volunteers receiving the heterologous booster vaccine was significantly higher (16% vs. 3%, *p* = 0.002). The serum SARS-CoV-2 spike Ig level decreases rapidly after a homologous booster dose, after a 14-day peak, while a gradual increase in the antibody level can be seen after a heterologous booster, Figure 1. Patient characteristics are outlined in Table 1.

### 3.2. Frequency of Adverse Reactions after 1st, 2nd and 3rd Dose of Vaccination

Adverse reactions occurred in 88/218 patients after the first vaccination, 87/218 after the 2nd vaccination, and 77/218 patients after the 3rd vaccination within 7 days after the dose. The total number of adverse reactions was 234 (1st dose), 252 (2nd dose) and 284 (3rd dose) after each vaccination. The frequency of fever (N, %) was 27 (13%), 22 (11%) and 36 (17%) after the 1st, 2nd, and 3rd vaccinations, respectively. The most common adverse reaction following the 2nd vaccination was local pain (47%), limb pain (47%), myalgia (36%) and fever (25%) while after the 3rd vaccination local pain was observed in 47%, limb pain in 47%, fever in 46% and chills in 34%. Fever was significantly more frequent after the 3rd vaccination than after the 2nd dose, *p* < 0.05.

### 3.3. Correlation of Antibody Titers with Adverse Reactions after the 2nd and 3rd Vaccinations

**Figure 2:**
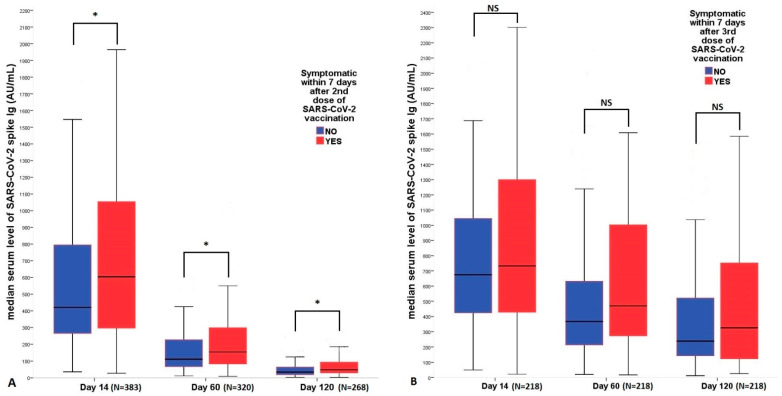
Correlation of antibody titers with syptomatic status after the 2nd and 3rd vaccinations. (**A**) Antibody response of symptomatic versus non-symptomatic patients at 14 (N = 383), 60 (N = 320) and 120 (N = 268) days after the 2nd vaccination, (**B**) after the 3rd dose (N = 218 at all time points). Definition of a symptomatic individual: A local or systemic adverse reaction occurring within 7 days after vaccination. Statistical analysis was performed using Mann–Whitney-U test in each group, respectively. NS, non-significant; SARS-CoV-2, severe acute respiratory syndrome coronavirus 2. * indicates *p* < 0.05.

The serum median SARS-CoV-2 Spike IgG level was significantly higher in the symptomatic group than in the asymptomatic group at all three time points after the second vaccination (Day 14, 60 and 120) Figure 2A. After the 3rd vaccination, this correlation disappeared, and we did not detect any significant difference between the serum SARS-CoV-2 spike Ig levels of the two groups Figure 2B. If only the serum levels of the volunteers receiving the homologous booster vaccine (N = 201) are examined, slightly significantly higher serum levels were observed in the symptomatic patients for the levels measured on days 14, 60 and 120 (*p* = 0.035, *p* = 0.049 and *p* = 0.170, respectively). In the case of volunteers who received a heterologous booster dose (N = 17), there was no difference in serum antibody levels between the symptomatic and the asymptomatic group during the studied period. In the case of fever appearing within 7 days after vaccination, significantly higher serum levels were found in the group with fever after both vaccinations, although the correlation is weaker after the 3rd vaccination, Table 2. We examined the correlations of serum S-IgG levels measured at four time points (Day 0, 14, 60 and 120) with demographic and clinical parameters. The Spearman r coefficient of correlation between all these parameters is presented in Table 3.

### 3.4. Factors Related to SARS-CoV-2 Positivity after the 3rd Vaccination

SARS-CoV-2 serum spike Ig levels measured before the 3rd dose and 14 days after the 3rd dose were significantly lower in patients who became COVID-19 positive within 120 days after the 3rd dose (before 3rd dose, COVID+: 64 [26–94] vs. COVID-: 79 [38–143], *p* = 0.036, 14 days after 3rd dose, COVID+: 527 [392–778] vs. 734 [461–1203], *p* = 0.015). COVID-19 positivity after 2nd dose and before 3rd dose (OR = 2.65; 95%CI = 1.05–6.69, *p* = 0.039) was independently associated with COVID-19 positivity within 120 days after booster vaccination, while fever after the 2nd dose (OR = 0.14; 95%CI 0.02–0.82; *p* = 0.028) was found to be independently associated with a reduction in the likelihood of COVID-19 positivity within 120 days after the booster dose. The SARS-CoV-2 spike Ig level immediately before the 3rd dose or 14 days after the 3rd vaccination did not prove to be an independent predictor of subsequent COVID positivity.

## 4. Discussion

Our previous study showed that adverse reactions after the second vaccination against SARS-CoV-2 are associated with a significantly higher serum SARS-CoV-2 spike Ig level for up to 6 months [5]. Other studies have also shown that specific IgG titers after basic BNT162b2 vaccination (2 doses) showed a positive correlation with the occurrence of adverse reactions [6,7]. In contrast, in the present study, we demonstrated that (i) the booster dose in the same cohort no longer shows any correlation between the appearance of adverse reactions and the subsequent antibody level, except a positive correlation with fever. However, this correlation is significantly weaker than observed after the second dose.

Other important findings of our study are the following: (ii) volunteers in the symptomatic group were younger, (iii) there were more symptomatic patients in the group receiving the heterologous booster vaccine, (iv) those who were symptomatic after the 1st and 2nd dose were more likely to be symptomatic after the 3rd vaccination as well, and (v) fever after 2nd dose was independently associated with a reduction in the likelihood of COVID-19 positivity after booster dose.

The adverse reactions after the booster vaccination were similar to those after the second dose according to recently published studies [4,11]. In our study, there was no difference between the adverse reactions observed after the 2nd and 3rd vaccination, although fever occurred more often after the 3rd dose. Next, the volunteers were grouped based on the booster vaccination into heterologous and homologous groups. After analysis, we did not find correlation between subsequent antibody levels and adverse reactions, although there were more symptomatic volunteers in the heterologous group. Previous studies have found a clear association between adverse events and subsequent antibody levels when using the basic homologous vaccination regime containing two doses [5,6,7]. After the 2nd COVID-19 vaccination, the number of memory B cells increased, and the number of memory T cells gradually decreased. However, with the third dose of the vaccine, the level of memory B cells continued to increase, while the levels of neutralizing antibodies and memory T cells returned to the level after the second vaccine administration [12]. mRNA COVID-19 vaccines generated functional memory B cells that increased from 3 to 6 months post-vaccination and further induced antigen-specific CD4^+^ and CD8^+^ T cells, and early CD4^+^ T cell responses [13]. Booster vaccinations could lead to a rebound in immune response against SARS-CoV-2 variants compared to a two-dose vaccination schedule. The broad responses may be due to the co-evolution of B cells in response to different variants, including SHM and memory B cell clonal turnover [14]. In a large cohort, heterologous boosters showed higher vaccine effectiveness than a homologous booster for laboratory-confirmed COVID-19 cases, hospitalization, admission to the ICU, and death, although there are no data on the distribution of adverse reactions [15]. A heterologous booster immunization strategy, primarily due to the differences in T-cell response, provides an immune response that may prove beneficial for long-term prevention [16]. Based on our results, our assumption is that the heterologous booster vaccine induces also induces additional immune mechanisms in the individual, which do not result in adverse reactions of the same strength and severity as in the case of the homologous vaccine. In contrast to the basic vaccination scheme, heterologous booster vaccines were also used in our study. Although the 3rd vaccine was heterologous in only a few patients in the present study, it could have still affected the positive correlation between antibody levels and side effects, which we also described earlier in the case of the basic vaccination scheme [5].

Although the difference in the increase in immunoglobulin levels between patients with and without fever is smaller after the third vaccination than after the second vaccination in our study, the booster effect of the vaccine remains evident: it triggered better sustained and higher levels of immunoglobulin compared to those observed following the second vaccination dose. While the second vaccination is part of the basic immunization, where the goal is to form immune memory and teach the immune system to respond to the spike protein, the booster dose is to strengthen the process, making these mechanisms permanent. Overall, it is likely that the different immune activation caused by the heterologous immunization is also associated with different adverse reactions, this could be one of the likely mechanisms why the adverse reactions after the 3rd vaccination did not show a correlation with the later antibody response. At the same time, the low number of cases might also explain why we were unable to demonstrate the clear correlation between adverse events and antibody levels that we observed in our previously examined population [5]. In addition, in contrast to the basic homologous vaccination strategy the booster vaccination also featured a heterologous version, which despite the low number of cases, may have influenced the number and quality of adverse reactions, given the different immunological mechanisms. A further study involving a higher number of volunteers would help to correctly interpret the differences between the quality and quantity of side effects and their correlation with subsequent antibody levels following the primary vaccination schedule and the booster vaccine that we observed in our current cohort of patients. Similar to a previous study [1], we also observed that systemic adverse reactions were reported more often by the younger volunteers than the older ones. This can be explained by the reduced strength of the inflammatory response in the elderly exposed to immune stress [17]. Another potential mechanism of this phenomenon is that older people show a greater tolerance for pain and disease symptoms acquired through life [18]. We found that after the heterologous booster vaccination, there were more symptomatic patients than after the homologous 3rd dose, which corresponds to what is described in the literature [19,20]. According to a recent study, spike-specific CD8 T-cell levels after heterologous vaccination were significantly higher than after homologous regimens [21]. Peripheral blood CD8+ T cells can be useful predictive markers of adverse events associated with the immune system during lung cancer therapy. The incidence of AEs was higher in the high CD8+ T cells group [22]. Another study also confirms that the severity of adverse reactions is associated with CD4+ and CD8+ T-cell response in mRNA-1273 vaccinated health care workers [23]. Based on these results, it is reasonable to assume that the immune processes triggered by the heterologous vaccine, which are different compared to those elicited by the homologous version, may be responsible for the more frequent adverse reactions.

Based on our data, it is evident that among the volunteers who experienced adverse reactions after the 3rd vaccination, there were significantly more patients who were also symptomatic after the 1st or 2nd dose. Our results are consistent with a recent study which found that adverse events after the second dose of the COVID-19 vaccine were predictors of more intense systemic adverse events notified within 7 days after the booster dose [24]. Finally, we found that fever after the 2nd dose was independently associated with a reduction in the likelihood of COVID-19 positivity after a booster dose. This can presumably be explained by the fact that fever following the second vaccination induces a stronger humoral immune response and the protective effect of the consequent higher antibody level prevails even after the booster vaccination.

Our study has some limitations. The number of patients receiving a heterologous booster dose is much lower than the number of patients receiving homologous vaccination, which may affect the strength of the statistical results. The follow-up period was only 3 months; a longer observation period would have allowed a better follow-up of the change in antibody levels of the respective groups. We only observed the humoral immune response and not the cellular immune response, so we can only explain the immunological changes that took place after the 3rd vaccination to a limited extent. At the same time, the strength of our study is that it points out that the type of booster vaccination (heterologous vs. homologous) probably causes different immune activation, which can also be inferred from the different vaccination reactions.

## Figures and Tables

**Table 1:** Characteristics of study population. The patients considered symptomatic if experienced adverse reactions within 7 days after vaccination vs. asymptomatic if no adverse reaction occurred after vaccination.

	Asymptomatic (N = 141)	Symptomatic (N = 77)	*p*-Value
Age, (mean ± SD)	49.6 ± 11	44 ± 11	0.001
Female, (N, %)	116 (82%)	57 (74%)	0.151
BMI, (mean ± SD)	28 ± 7	27 ± 5	0.474
Smoking, (N, %)	45 (33%)	23 (32%)	0.947
Time lag between 2nd and 3rd vaccine dose, days, (mean ± SD)	250 ± 45	247 ± 41	0.827
Pfizer BioNTech vaccine, 3rd dose, (N, %)	136 (97%)	65 (84%)	0.002
Hypertension, (N, %)	40 (29%)	17 (23%)	0.359
Diabetes, type II, (N,%)	10 (7%)	5 (7%)	0.904
Allergy, (N, %)	32 (23%)	28 (38%)	0.022
Autoimmune disease, (N, %)	10 (7%)	3 (4%)	0.361
Symptomatic after 1st dose, (N, %)	43 (31%)	43 (56%)	<0.001
Symptomatic after 2nd dose, (N, %)	40 (28%)	45 (58%)	<0.001
SARS-CoV-2 positivity before 3rd dose, (N, %)	45 (32%)	17 (22%)	0.124
SARS-CoV-2 positivity after 3rd dose, (N, %)	31 (22%)	23 (30%)	0.197

Abbreviations: BMI, body mass index; SD, standard deviation; SARS-CoV-2, severe acute respiratory syndrome coronavirus 2; N, number.

**Table 2:** Changes in serum SARS-CoV-2 spike Ig levels on Days 14, 60 and 120 after the 2nd and 3rd vaccination, depending on whether fever occurred within 7 days after the vaccination. Number of patients with fever (N,%) after 3rd dose: 41/218 (19%).

Fever	Minimum	25%	Median	75%	Maximum	*p*-Value
			After 2nd dose			
			Day 14 (N = 383)			
+ (N = 56)	266	689	986	1402	7785	<0.001
− (N = 327)	27	262	442	810	655	
			Day 60 (N = 320)			
+ (N = 49)	96	164	274	457	998	<0.001
− (N = 271)	9	70	123	237	655	
			Day 120 (N = 268)			
+ (N = 45)	28	49	76	148	251	<0.001
− (N = 223)	2	19	36	68	379	
			After 3rd dose			
			Day 14 (N = 218)			
+ (N = 41)	47	388	955	1570	3209	0.045
− (N = 177)	22	425	663	1014	5948	
			Day 60 (N = 218)			
+ (N = 41)	107	331	790	1190	4117	0.002
− (N = 177)	17	208	379	670	7101	
			Day 120 (N = 218)			
+ (N = 41)	56	260	494	815	3005	0.014
− (N = 177)	12	124	240	541	2706	

Abbreviations: SARS-CoV-2, severe acute respiratory syndrome coronavirus 2; Ig, immunoglobuline.

**Table 3:** Variables associated with levels of S-IgG (AU/mL) in cross-sectional analysis. Day 0, S-IgG measurements immediately before 3rd dose; Day 14, Day 60, and Day 120 S-IgG, S-IgG measurements on 14, 60 and 120 days after 3rd dose. Values are Spearman correlation coefficients. * *p* < 0.05, ** *p* < 0.001. S-IgG, anti-spike immunglobulin; AU, arbitrary unit; COVID+, confirmed corona virus disease-19, mRNS, messenger ribonucleic acid.

Variable	Day 0 S-IgG	Day 14 S-IgG	Day 60 S-IgG	Day 120 S-IgG
**Age**	−0.190 **	−0.030	−0.028	−0.089
**Smoking**	−0.163 *	−0.050	−0.128	−0.080
**Gender**	0.013	0.158 *	0.134 *	0.149 *
**mRNS type vaccine**	−0.149 *	−0.317 **	−0.032	0.031
**COVID+ before 1st dose**	0.293 **	0.004	−0.024	−0.042
**COVID+between 2nd and 3rd dose**	0.144 *	0.174 *	0.204 **	0.116
**NSAID**	−0.163 *	−0.140 *	−0.115	−0.145 *
**Hyperlipidaemia**	−0.138 *	−0.166 *	−0.157 *	−0.082
**Chills after 2nd dose**	0.209 **	0.189 **	0.138	0.091
**Fever after 2nd dose**	0.281 **	0.261 **	0.226 **	0.172 **
**Chills after 3rd dose**	N/A	0.138 *	0.203 **	0.143
**Fever after 3rd dose**	N/A	0.145 *	0.262 **	0.189 **
**Use of beta blocker**	−0.169 *	−0.054	−0.123	−0.019
**Time lag between 2nd and 3rd dose**	−0.097	0.012	0.118	0.216 **

## Data Availability

All relevant data are within the manuscript.

## References

[1] Polack F.P., Thomas S.J., Kitchin N., Absalon J., Gurtman A., Lockhart S., Perez J.L., Pérez Marc G., Moreira E.D., Zerbini C. (2020). Safety and Efficacy of the BNT162b2 mRNA Covid-19 Vaccine. N. Engl. J. Med..

[2] Baden L.R., El Sahly H.M., Essink B., Kotloff K., Frey S., Novak R., Diemert D., Spector S.A., Rouphael N., Creech C.B. (2021). Efficacy and Safety of the mRNA-1273 SARS-CoV-2 Vaccine. N. Engl. J. Med..

[3] Dighriri I.M., Alhusayni K.M., Mobarki A.Y., Aljerary I.S., Alqurashi K.A., Aljuaid F.A., Alamri K.A., Mutwalli A.A., Maashi N.A., Aljohani A.M. (2022). Pfizer-BioNTech COVID-19 Vaccine (BNT162b2) Side Effects: A Systematic Review. Cureus.

[4] Menni C., May A., Polidori L., Louca P., Wolf J., Capdevila J., Hu C., Ourselin S., Steves C.J., Valdes A.M. (2022). COVID-19 vaccine waning and effectiveness and side-effects of boosters: A prospective community study from the ZOE COVID Study. Lancet Infect. Dis..

[5] Kanizsai A., Molnar T., Varnai R., Zavori L., Tőkés-Füzesi M., Szalai Z., Berecz J., Csecsei P. (2022). Fever after Vaccination against SARS-CoV-2 with mRNA-Based Vaccine Associated with Higher Antibody Levels during 6 Months Follow-Up. Vaccines.

[6] Levy I., Levin E.G., Olmer L., Regev-Yochay G., Agmon-Levin N., Wieder-Finesod A., Indenbaum V., Herzog K., Doolman R., Asraf K. (2022). Correlation between Adverse Events and Antibody Titers among Healthcare Workers Vaccinated with BNT162b2 mRNA COVID-19 Vaccine. Vaccines.

[7] Koike R., Sawahata M., Nakamura Y., Nomura Y., Katsube O., Hagiwara K., Niho S., Masuda N., Tanaka T., Sugiyama K. (2022). Systemic Adverse Effects Induced by the BNT162b2 Vaccine Are Associated with Higher Antibody Titers from 3 to 6 Months after Vaccination. Vaccines.

[8] Wise J. (2021). COVID-19: People who have had infection might only need one dose of mRNA vaccine. BMJ (Clin. Res. Ed.).

[9] Krammer F., Srivastava K., Simon V. (2021). Robust spike antibody responses and increased reactogenicity in seropositive individuals after a single dose of SARS-CoV-2 mRNA vaccine. medRxiv.

[10] Manisty C., Otter A.D., Treibel T.A., McKnight Á., Altmann D.M., Brooks T., Noursadeghi M., Boyton R.J., Semper A., Moon J.C. (2021). Antibody response to first BNT162b2 dose in previously SARS-CoV-2-infected individuals. Lancet.

[11] Chu L., Vrbicky K., Montefiori D., Huang W., Nestorova B., Chang Y., Carfi A., Edwards D.K., Oestreicher J., Legault H. (2022). Immune response to SARS-CoV-2 after a booster of mRNA-1273: An open-label phase 2 trial. Nat. Med..

[12] Mise-Omata S., Ikeda M., Takeshita M., Uwamino Y., Wakui M., Arai T., Yoshifuji A., Murano K., Siomi H., Nakagawara (2022). Memory B Cells and Memory T Cells Induced by SARS-CoV-2 Booster Vaccination or Infection Show Different Dynamics and Responsiveness to the Omicron Variant. J. Immunol..

[13] Goel R.R., Painter M.M., Apostolidis S.A., Mathew D., Meng W., Rosenfeld A.M., Lundgreen K.A., Reynaldi A., Khoury D.S., Pattekar A. (2021). mRNA vaccines induce durable immune memory to SARS-CoV-2 and variants of concern. Science.

[14] Gaebler C., Wang Z., Lorenzi J.C.C., Muecksch F., Finkin S., Tokuyama M., Cho A., Jankovic M., Schaefer-Babajew D., Oliveira T.Y. (2021). Evolution of antibody immunity to SARS-CoV-2. Nature.

[15] Jara A., Undurraga E.A., Zubizarreta J.R., González C., Pizarro A., Acevedo J., Leo K., Paredes F., Bralic T., Vergara V. (2022). Effectiveness of homologous and heterologous booster doses for an inactivated SARS-CoV-2 vaccine: A large-scale prospective cohort study. Lancet. Glob. Health.

[16] Atmar R.L., Lyke K.E., Deming M.E., Jackson L.A., Branche A.R., El Sahly H.M., Rostad C.A., Martin J.M., Johnston C., Rupp R.E. (2022). Homologous and Heterologous Covid-19 Booster Vaccinations. N. Engl. J. Med..

[17] El Yousfi M., Mercier S., Breuillé D., Denis P., Papet I., Mirand P.P., Obled C. (2005). The inflammatory response to vaccination is altered in the elderly. Mech. Ageing Dev..

[18] Hervé C., Laupèze B., Del Giudice G., Didierlaurent A.M., Tavares Da Silva F. (2019). The how’s and what’s of vaccine reactogenicity. NPJ Vaccines.

[19] Powell A.A., Power L., Westrop S., McOwat K., Campbell H., Simmons R., Ramsay M.E., Brown K., Ladhani S. (2021). Real-world data shows increased reactogenicity in adults after heterologous compared to homologous prime-boost COVID-19 vaccination, March-June 2021, England. Eur. Commun. Dis. Bull..

[20] Warkentin L., Zeschick N., Kühlein T., Steininger P., Überla K., Kaiser I., Gall C., Sebastião M., Hueber S. (2022). Reactogenicity after heterologous and homologous COVID-19 prime-boost vaccination regimens: Descriptive interim results of a comparative observational cohort study. BMC Infect. Dis..

[21] Schmidt T., Klemis V., Schub D., Mihm J., Hielscher F., Marx S., Abu-Omar A., Ziegler L., Guckelmus C., Urschel R. (2021). Immunogenicity and reactogenicity of heterologous ChAdOx1 nCoV-19/mRNA vaccination. Nat. Med..

[22] Wu K., Yang S., Li X., Xia B., Ma S., Chen X. (2021). MA09.03 Peripheral CD8+ T Cells Predicts Immune-Related Adverse Events and Survival in Advanced Non-Small Cell Lung Cancer Treated With Immunotherapy. J. Thorac. Oncol..

[23] Klingel H., Lauen M., Krüttgen A., Imöhl M., Kleines M. (2022). Severity of adverse reactions is associated with T-cell response in mRNA-1273 vaccinated health care workers. Clin. Exp. Vaccine Res..

[24] Stosic M., Milic M., Markovic M., Kelic I., Bukumiric Z., Veljkovic M., Kisic Tepavcevic D., Saponjic V., Plavsa D., Jovanovic S. (2022). Immunogenicity and Reactogenicity of the Booster Dose of COVID-19 Vaccines and Related Factors: A Panel Study from the General Population in Serbia. Vaccines.

